# Supplementary material for: The prevalence of protein misfolding as a mechanism for hereditary deafness
Source: PLoS Genet. 2026 Sep 30;22(9):e1012085. doi: 10.1371/journal.pgen.1012085 (PMC13641712; doi:10.1371/journal.pgen.1012085)
Supplement: Supplementary file 1 [file pgen.1012085.s001.docx]

**S1 Appendix**

*Description of Polynomial Fit and Variant Prioritization*

The biophysics-informed thresholds were determined using MATLAB contour and polynomial fit functions. We used the biophysics-informed likelihoods to calculate posterior values for 100 (10x10) discrete bins. These bins are not tolerance-based but grouped by score to overcome scores without enough data available to calculate a likelihood ratio. Using MATLAB’s contour function, we input the midpoints of the bins for each score, the posterior probability for each bin (a 10x10 matrix), and the posterior probability for each evidence level. The contour function provides data points along which the contour falls for the specified posterior. The x- and y-coordinates from the contour function are inputs to a polynomial fit function with the order chosen to most closely match the visualized contour for that evidence level. For REVEL x $\Delta\Delta G_{Fold}$, strong and very strong evidence followed a linear fit and supporting and moderate followed a parabolic fit. CADD x $\Delta\Delta G_{Fold}$ follows an exclusively parabolic fit for all evidence levels. A different equation was calculated for each prior value to result in four fit curves for the tolerance-based thresholds. We then calculate an expected $\Delta\Delta G_{Fold}$ for a given REVEL or CADD value. If the actual $\Delta\Delta G_{Fold}$ is greater than the expected $\Delta\Delta G_{Fold}$, it is considered above the threshold. The goal was to establish a simple, easy-to-apply threshold for prioritization.

For example, we will evaluate a VUS using the REVEL x $\Delta\Delta G_{Fold}$ tolerance-based method. The *ACTG1* variant p.(Ile357Met) has a o/e of 0.43, a REVEL score of 0.84, and a $\Delta\Delta G_{Fold}$ of 1.4 kcal/mol. We first select the fit curve from the intolerant bin. This results in the equation of a line:

$$expected \Delta\Delta G_{Fold}= -9.43 \left( REVEL \right)+8.17$$

We evaluate Equation 1 based on the variant’s REVEL score to calculate an expected $\Delta\Delta G_{Fold}$.

$$expected \Delta\Delta G_{Fold}= -9.43 \left( 0.84 \right)+8.17$$

$$expected \Delta\Delta G_{Fold}= 0.25$$

Since expected $\Delta\Delta G_{Fold}$ ≤ actual $\Delta\Delta G_{Fold}$ of 1.4 kcal/mol, this variant would be prioritized and given the VUS designation. For the tolerance-based method, the equation depends on the tolerance of the gene. For example, this same variant evaluated with the tolerant threshold:

$$expected \Delta\Delta G_{Fold}= -10.07 \left( 0.84 \right)+9.93$$

$$expected \Delta\Delta G_{Fold}= 1.47$$

In this case, a variant with the same $\Delta\Delta G_{Fold}$ and REVEL score would not be prioritized if it has a tolerance score of 1.0 and above.

*Protein Folding Analysis*

We calculated thresholds for $\Delta\Delta G_{Fold}$ classification prediction alone. The best performance was seen for the tolerance-based method with an accuracy of 4.3%, sensitivity of 16.9%, and specificity of 2.5%. With these results, we can conclude $\Delta\Delta G_{Fold}$ alone does not sufficiently predict classifications.

*Benign Protein Folding-Informed Analysis*

Here, the goal is to determine if $\Delta\Delta G_{Fold}$ combined with CADD or REVEL could provide additional evidence for predicting benign variants in the DVD. We used the same Bayesian framework described in the method sections, but with benign prior probability and likelihood ratio calculations. The benign priors were calculated as 1-prior odds of pathogenicity and the likelihood ratios are the reciprocal of the pathogenic likelihood ratio. We then followed the same procedure to determine what scores merited a very strong evidence redesignation (posterior of 1.0). For the DVD prior and tolerance-based priors, we did not reach very strong evidence with the inclusion of $\Delta\Delta G_{Fold}$. $\Delta\Delta G_{Fold}$ is not typically used to deprioritize variants as benign because it is only testing one possible mechanism of pathogenicity (protein misfolding) while variants can cause many different downstream effects (*i.e.* disruption of protein-protein binding). Therefore, we avoid using $\Delta\Delta G_{Fold}$ to label benign variants.

*AlphaMissense with Bayesian Thresholds for Evidence Types*

AlphaMissense is a deep learning network trained to provide a continuous score of pathogenicity for missense variants(1). The scores range from 0 to 1 with 1 being more likely to be deleterious. A limitation of AlphaMissense is that new scores cannot be calculated because the training weights have not been released. AlphaMissense is based on the AlphaFold2 proteome wide database of structures. However, this focuses mainly on canonical isoforms and only a limited of genes offer additional isoforms. In the case of the DVD, there are ~20,000 labeled variants without AlphaMissense scores due to a non-canonical isoform that was not covered by the AlphaFold2 database. This means a limited set of variants is available to perform an analysis comparable to what has been presented for CADD and REVEL.

Here, we evaluate AlphaMissense for its performance on the DVD with the Bayesian Framework used to evaluate CADD and REVEL, albeit for a subset of labeled variants in the DVD. Using the DVD prior probability of pathogenicity 12.6%, AlphaMissense has an accuracy of 0.10, a sensitivity of 0.70, and a specificity of 0.0 for strong thresholds. REVEL has an accuracy of 0.52, a sensitivity of 0.79, and a specificity of 0.48 for the same reduced dataset. Evaluating AlphaMissense using the tolerance-based binning, the accuracy is 0.10, the sensitivity is 0.65, and specificity of 0.0. AlphaMissense does not perform better than REVEL and CADD when translating scores to ACMG criteria in the DVD.

Since AlphaMissense implicitly includes information regarding protein structure, it is reasonable to compare our REVEL x $\Delta\Delta G_{Fold}$ model to AlphaMissense alone. AlphaMissense with the DVD prior probability of pathogenicity (12.6%) or the tolerance-based priors does not reach very strong evidence (posterior of 98%). REVEL x $\Delta\Delta G_{Fold}$ does reach very strong evidence making it a more useful method for prioritizing VUSs for further investigation.

We acknowledge AlphaMissense’s given classifications are often accurate to the labeled data. For the DVD, this does result in 92% accuracy, 75% sensitivity, and 94% specificity. However, these labels are not consistent with ACMG criteria. When the AlphaMissense scores are used to define the evidence thresholds needed for ACMG criteria, a significant drop in accuracy and specificity is seen. The AlphaMissense score needed to reach strong evidence levels by ACMG criteria with *in silico* tools results in more stringent cutoffs for pathogenic and benign classifications than those used to determine the AlphaMissense classifications. Additionally, AlphaMissense performance shows varying performance on other datasets, while alternative tools have similar or better performance, including REVEL(2-7). This is seen in its reduced sensitivity for the DVD dataset. Considering AlphaMissense’s modest performance on DVD labeled variants, the lack of maintenance for the tool, and the missing data for DVD isoforms, AlphaMissense is not a suitable replacement for CADD or REVEL in the context of the DVD.

*Comparing REVEL and REVEL x* $\Delta\Delta G_{Fold}$ *to Experimental KCNQ4 Dataset*

(8). We first examine the performance of variant effect prediction tools across all 4085 SNVs, followed by further examination of the 516 reassessed variants. We calculated accuracy, sensitivity, and specificity for 60% posterior and 98% posterior AlphaMissense, REVEL, and REVEL x ∆∆$G_{Fold}$ thresholds against MAVE data by assigning labels based on normalized current values. Variants with a normalized current less than 0.54 (set by Zheng et al) were labeled pathogenic while any variant greater than 0.54 was labeled benign. We exclude five variants in the starting methionine, which is a start loss mutation rather than a missense mutation. For comparison, we also perform the same evaluation using labeled DVD variants. The results are summarized in Table A.

|  | **Evidence Strength** | **Method** | **Accuracy** | **Sensitivity** | **Specificity** |
| --- | --- | --- | --- | --- | --- |
| **MAVE Variant Labels** | Posterior 60% | AM | 0.1850 | 0.8260 | 0.0 |
|  |  | REVEL | 0.2034 | 0.9092 | 0.0 |
|  |  | REVEL x ∆∆$G_{Fold}$ | 0.2012 | 0.9034 | 0.0 |
|  | Posterior 98% | AM | 0.0 | 0.0 | 0.0 |
|  |  | REVEL | 0.0534 | 0.2385 | 0.0 |
|  |  | REVEL x ∆∆$G_{Fold}$ | 0.1174 | 0.5263 | 0.0 |
| **DVD Variant Labels** | Posterior 60% | AM | 0.1765 | 0.8125 | 0.0 |
|  |  | REVEL | 0.1991 | 0.8958 | 0.0058 |
|  |  | REVEL x ∆∆$G_{Fold}$ | 0.1946 | 0.8958 | 0.0 |
|  | Posterior 98% | AM | 0.0 | 0.0 | 0.0 |
|  |  | REVEL | 0.0950 | 0.4168 | 0.0 |
|  |  | REVEL x ∆∆$G_{Fold}$ | 0.1538 | 0.7083 | 0.0 |

**Table A. Calculated accuracy, sensitivity, and specificity for AlphaMissense (AM), REVEL, and REVEL x ∆∆**$\boldsymbol{G}_{\boldsymbol{Fold}}$ **for the *KCNQ4* MAVE dataset and KCNQ4 DVD labeled variants**. We include both values with a posterior probability of pathogenicity of 60% and 98% to show improvement when including ∆∆$G_{Fold}$.

While we still see difficulty predicting benign variants (specificity of ~0.0 for all methods), REVEL has a slightly higher sensitivity at a posterior of 60%. However, when increasing to a posterior of 98%, REVEL x ∆∆$G_{Fold}$ has the highest sensitivity for both the MAVE and DVD labels. AlphaMissense thresholds do not reach a posterior value of 98%, meaning it is unable to predict pathogenic variants to this level of evidence. This is in line with our conclusion that a posterior of 98% for REVEL x ∆∆$G_{Fold}$ is the best method for prioritizing variants as pathogenic with the highest possible evidence level. The accuracy for all methods is below 25% because no method can accurately predict benign variants, which remains a challenge in the field.

Due to the additional experimental evidence, a subset of 112 variants reported in the *KNCQ4* study were reclassified from VUS (based on ClinVar) to likely pathogenic/pathogenic (25 variants) or likely benign/benign (87 variants). We evaluate the prioritized variants from our model against this subset of newly reclassified variants due to their clinical significance and to validate our method against a set of variants not included in the building of our model. The VUS reclassified to likely pathogenic/pathogenic will be referred to as VUS_LP and VUS reclassified to likely benign/benign will be referred to as VUS_LB. Of the 112 variants, 43 have a DVD classification and will be excluded from further analysis because they were included in the building of our model. Additionally, 5 variants in the experimental set are not currently included in the DVD and therefore excluded from the analysis. This leaves 14 VUS_LP and 50 VUS_LB.

First, we compare the variants prioritized with REVEL and REVEL x $\Delta\Delta G_{Fold}$ to the 14 VUS_LP. Four variants reached the REVEL value (0.947) needed for very strong evidence of pathogenicity in a gene of average tolerance (*KCNQ4* o/e is 0.89). With REVEL x $\Delta\Delta G_{Fold}$, eight variants in the VUS_LP set were prioritized. While this test set is small, double the number of variants are prioritized when using REVEL x $\Delta\Delta G_{Fold}$ compared to REVEL alone. Additionally, of the 50 VUS_LB variants, only one was prioritized with REVEL x $\Delta\Delta G_{Fold}$. By comparing our prioritized variants to newly classified variants, we validate our method on classified variants not included in the building of our model and reiterate the merits of including ∆∆$G_{Fold}$ with REVEL to better select VUS as likely to be pathogenic.

While this *KCNQ4* study consists of an impressive experimental analysis of 4085 variants, there are some limitations that should be noted. The experiments were performed in hamster ovarian cells transfected with wildtype and mutant *KCNQ4* plasmids. Although hamster ovarian cells are a suitable model for whole-cell patch-clamp experiments, it is uncertain if these results are fully consistent with the regulation and function of *KCNQ4* in human hair cells. Furthermore, this MAVE data is limited to PS3 supporting evidence, which is unable to provide a definitive clinical classification based on ACMG criteria.

|  |  | Likely/Benign | | Likely/Pathogenic | | VUS > 98% | |
| --- | --- | --- | --- | --- | --- | --- | --- |
|  |  | Uniform | Tolerance-Based | Uniform | Tolerance-Based | Uniform | Tolerance-Based |
| CADD x $\Delta\Delta G_{Fold}$ | Intolerant | 6 | 19 | 262 | 515 | 2,424 | 5,043 |
|  | Average Tolerance | 21 | 18 | 573 | 513 | 6,750 | 5,816 |
|  | Tolerant | 5 | 0 | 282 | 210 | 1,939 | 1,343 |
|  | X-linked/MT | 2 | 2 | 74 | 74 | 585 | 585 |
| **Total** | **CADD** | **-** | **3** | **-** | **96** | **-** | **615** |
|  | **CADD x** $\boldsymbol{\Delta\Delta}\boldsymbol{G}_{\boldsymbol{Fold}}$ | **34** | **39** | **1,191** | **1,312** | **11,698** | **12,787** |

**Table B. Variants for each label that are prioritized by CADD x** $\boldsymbol{\Delta\Delta}\boldsymbol{G}_{\boldsymbol{Fold}}$**.** The classification of variants with very strong evidence from CADD alone and CADD x $\Delta\Delta G_{Fold}$ for using DVD prior and tolerance-based priors. CADD alone only reached very strong evidence in the intolerant bin with its associated prior probability of pathogenicity. Therefore, we only include the CADD alone numbers in the total row.

| **Gene** | **Variant** | **REVEL** | $\boldsymbol{\Delta\Delta}\boldsymbol{G}_{\boldsymbol{Fold}}$ **(kcal/mol)** | **Patient Info** | **Structural Analysis** |
| --- | --- | --- | --- | --- | --- |
| TECTA | Cys2052Tyr | 0.909 | 2.0 | Congenital severe bilateral SNHL, family history of hearing loss in mom, maternal grandfather |   Cys2052 has an annotated disulfide bond with Cys2045 in Uniprot and mutating to tyrosine breaks this bond, affecting that protein folding stability. |
|  | Tyr1900Cys | 0.883 | 2.6 | Moderate bilateral SNHL, family history of HL in mom, maternal grandmother |   Cys1086 and Cys1898 have an annotated disulfide bond and mutating from tyrosine to cysteine could disrupt the established bond between these two cysteines, changing the folding stability of the protein. |
|  | Cys696Ser | 0.753 | 3.4 | Early childhood mild bilateral mixed HL |   Cys696 is close to 3 other cysteines (Cys690, Cys705, and Cys671), because alpha tectorin is in the extracellular matrix, it is in an oxidizing environment which would make cysteines reactive and likely form disulfide bonds to stabilize the fold, a serine would break this bonding motif. |
| MYO6A | Leu1086Pro | 0.847 | 4.1 | Congenital mild-to-moderate bilateral SNHL, sibling with HL |   Leu1086 is in an alpha helix and proline is a known secondary structure and alpha helix breaker, causing protein misfolding. |
|  | Leu120Pro | 0.895 | 2.1 | Early childhood mild bilateral SNHL, family history of HL in father, paternal grandfather |   Leu120 is in a disordered region between two alpha helices, it is likely the turn between the helices is necessary for structure/function and proline promotes disordered regions, most likely leading to too much disorder and causes protein misfolding |
|  | Leu74Pro | 0.949 | 4.5 | Early childhood bilateral mild-to-moderate SNHL, family history of sibling, mom, maternal aunt, maternal grandfather with HL |   Leu74 is in an alpha helix, proline is a known helix breaker and will promote protein misfolding |
| TMPRSS3 | Asp400Glu | 0.916 | 0.8 | Early childhood bilateral SNHL progressive to severe-to-profound |   Asp400 is buried and forms hydrogen bonds with the backbone, glutamate is larger than aspartate and could cause steric clashes with the backbone, leading to protein misfolding |
| MYO15A | Tyr2282Cys | 0.909 | 3.2 | Early childhood onset bilateral profound SNHL, sibling with similar HL | While this variant was selected by our prioritzation methods, there is not a clear structural reason the folding free energy difference is high. |
| OTOF | Met966Arg | 0.785 | 2.1 |  |   Met966 (orange) is a hydrophobic residue surrounded by other hydrophobic residues (cyan). Mutating to hydrophillic destabilizes the favorable interaction of the surrounding residues. Opposite Ile1573Thr |
| ADGRV1 | Tyr1486Cys | 0.795 | 3.0 | Congenital normal downsloping to moderate bilateral SNHL |   Tyr1486 is surface exposed, ADGRV1 is in the extracellular matrix (an oxidixing environment). Mutating to cysteine in an oxidizing environment would make the cysteine very reactive, likely causing the protein misfold. |
| GATA3 | Pro354Leu | 0.968 | 0.9 | Early childhood onset asymmetric SNHL; bilateral posterior semicricular canal hypoplasia, bilateral malleoincudal fusion; ectopic ureter |   GATA3 is intrinsically disordered and prolines promote disorder. Mutating from proline to leucine could cause the alpha helices on either side to connect, making the proline favored. |
| STRC | Leu1414 Pro | 0.614 | 4.1 | Childhood onset moderate bilateral SNHL |   Leu1414 is in an alpha helix, proline is a known helix breaker, causing protein misfolding. |

**Table C. Describes patient variants selected by REVEL x** $\boldsymbol{\Delta\Delta}\boldsymbol{G}_{\boldsymbol{Fold}}$ **and the probable cause of selection.** There are four variants that are known pathogenic. One variant (*OTOF* p.Met966Arg) occurs opposite to a known pathogenic variant (*OTOF* p.Ile1573Thr).

| CADD | | Range | | | [0, 4) | [4, 8) | | [8, 12) | | | [12, 16) | | [16, 20) | | [20, 24) | | [24, 28) | | [28, 32) | | [32, 36) | | [36, 40] |
| --- | --- | --- | --- | --- | --- | --- | --- | --- | --- | --- | --- | --- | --- | --- | --- | --- | --- | --- | --- | --- | --- | --- | --- |
|  |  | ${LR}_{P}$ | | | 0.0242 | 0.0356 | | 0.0378 | | | 0.0481 | | 0.1144 | | 0.8976 | | 20.7539 | | 61.0575 | | 98.9463 | | 81.5486 |
|  |  | ${LR}_{B}$ | | | 41.3307 | 28.0651 | | 26.4581 | | | 20.7959 | | 8.7387 | | 1.1141 | | 0.0482 | | 0.0164 | | 0.0101 | | 0.0123 |
| REVEL | | Range | | | [0, 0.1) | [0.1, 0.2) | | [0.2, 0.3) | | | [0.3, 0.4) | | [0.4, 0.5) | | [0.5, 0.6) | | [0.6, 0.7) | | [0.7, 0.8) | | [0.8, 0.9) | | [0.9, 1.0] |
|  |  | ${LR}_{P}$ | | | 0.0211 | 0.0860 | | 0.2005 | | | 0.4753 | | 1.7761 | | 5.4862 | | 15.3796 | | 37.9631 | | 133.1624 | | 696.1288 |
|  |  | ${LR}_{B}$ | | | 47.3040 | 11.6321 | | 4.9868 | | | 2.1037 | | 0.5630 | | 0.1823 | | 0.0650 | | 0.0263 | | 0.0075 | | 0.0014 |
| ∆∆$G_{Fold}$ | | Range | | | [0, 0.65) | [0.65, 1.30) | | [1.30, 1.95) | | | [1.95, 2.60) | | [2.60, 3.25) | | [3.25, 3.90) | | [3.90, 4.55) | | [4.55, 5.20) | | [5.20, 5.85) | | [5.85, 6.50] |
|  |  | ${LR}_{P}$ | | | 0.6053 | 2.8239 | | 5.8487 | | | 10.4236 | | 18.6176 | | 62.4628 | | 143.4330 | | 182.7614 | | 541.3439 | | 101.7912 |
|  |  | ${LR}_{B}$ | | | 1.6521 | 0.3541 | | 0.1710 | | | 0.0959 | | 0.0537 | | 0.0160 | | 0.0070 | | 0.0055 | | 0.0018 | | 0.0098 |
|  |  | | |  | | |  | |  |  | |  | |  | |  | |  | |  | |  | |

**Table D. The likelihood ratios for pathogenic and benign variants.** This includes ratios for ranges of predicted folding free energy difference, REVEL, and CADD.

**Fig A.** Distribution of o/e scores for the genome (pink) and DVD genes (blue).

**Fig B.** **Tolerance-based genetic tool threshold plots for benign and pathogenic thresholds.** Panel A shows the CADD thresholds for the intolerant, average tolerance, and tolerant bin. Panel B shows the REVEL thresholds for the intolerant, average tolerance, and tolerant bin.

**Fig C. Scatterplots of CADD x** $\boldsymbol{\Delta\Delta}\boldsymbol{G}_{\boldsymbol{Fold}}$ **for strong evidence.** Panel A shows the CADD x $\Delta\Delta G_{Fold}$ very strong evidence thresholds for classified variants. Panel B shows CADD x $\Delta\Delta G_{Fold}$ with VUSs above the 98% posterior are colored red.

**Fig D. Scatterplots of** $\boldsymbol{\Delta\Delta}\boldsymbol{G}_{\boldsymbol{Fold}}$ **vs CADD for missense variants colored by label.** The thresholds for very strong evidence in the intolerant, average tolerance, and tolerant bins. Panel A shows only classified variants for CADD x $\Delta\Delta G_{Fold}$ with the four thresholds of evidence for intolerant genes. Panel B shows the CADD x $\Delta\Delta G_{Fold}$ thresholds for prioritized variants in the intolerant bins. Panel C and Panel D show CADD x $\Delta\Delta G_{Fold}$ show the thresholds for the average tolerance bins. Panel E and Panel F show CADD x $\Delta\Delta G_{Fold}$ thresholds for the tolerant bin.

**Figure E. A bar chart of the percentage of LP/P with at least 1 kcal/mol** $\boldsymbol{\Delta\Delta}\boldsymbol{G}_{\boldsymbol{Fold}}$ **per gene.** This chart includes genes with at least 10 pathogenic variants.

**References**

1.	Cheng J, Novati G, Pan J, Bycroft C, Zemgulyte A, Applebaum T, et al. Accurate proteome-wide missense variant effect prediction with AlphaMissense. Science. 2023;381(6664):1303-+.

2.	Fortier N. Golden Helix2023. [cited 2026].

3.	Lindquist M, Darrah S, Stafie ST, Mustafi D. Benchmarking AlphaMissense against ClinVar for Diagnostic Interpretation of Missense Variants in Inherited Retinal Diseases. Ophthalmol Sci. 2026;6(2):100997.

4.	Pillai J, Liu S, Sung K, Shi L, Wu C. Benchmarking AlphaMissense pathogenicity predictions against APP, PSEN1, and PSEN2 variants of unknown significance. Biochem Biophys Rep. 2025;42:102049.

5.	Ruiz M, Ochoa JP, Migoyo-Bettoni C, de la Barrera J, Delrio-Lorenzo A, Fernandez-Rojo MA, et al. Performance of AlphaMissense and Other In Silico Predictors to Determine Pathogenicity of Missense Variants in Sarcomeric Genes. Circ Genom Precis Med. 2025;18(2):e004922.

6.	Ryan-Phillips F, Henehan L, Ramdas S, Palace J, Beeson D, Dong YY. Assessing the Utility of ColabFold and AlphaMissense in Determining Missense Variant Pathogenicity for Congenital Myasthenic Syndromes. Biomedicines. 2024;12(11).

7.	Zhuo X, Bi X, Aggarwal V, Liao J. Calibration and evaluation of machine-learning algorithms for missense variant classification under ACMG/ClinGen recommendations. Genet Med. 2026:102606.

8.	Zheng H, Yan X, Li G, Lin H, Deng S, Zhuang W, et al. Proactive functional classification of all possible missense single-nucleotide variants in KCNQ4. Genome Res. 2022;32(8):1573-84.

9.	Benjamin J. Livesey JAM. Why variant effect predictors and multiplexed assays agree and

disagree bioRxiv. 2025.

10.	Vivekanandam V, Ellmers R, Jayaseelan D, Houlden H, Mannikko R, Hanna MG. In silico versus functional characterization of genetic variants: lessons from muscle channelopathies. Brain. 2023;146(4):1316-21.

11.	Brown DA. Kv7 (KCNQ) potassium channels that are mutated in human diseases. J Physiol. 2008;586(7):1781-3.
